# Use of Digital Health Interventions in Sub-Saharan Africa for Health Systems Strengthening Over the Last 10 Years: A Scoping Review Protocol

**DOI:** 10.3389/fdgth.2022.874251

**Published:** 2022-05-06

**Authors:** Hillary Kipruto, Derrick Muneene, Benson Droti, Violet Jepchumba, Chukwudi Joseph Okeibunor, Juliet Nabyonga-Orem, Humphrey Cyprian Karamagi

**Affiliations:** ^1^WHO Regional Office for Africa, Inter Country Support Team for Eastern and Southern Africa, Harare, Zimbabwe; ^2^WHO Headquarters, Geneva, Switzerland; ^3^Universal Health Coverage Life Course Cluster, WHO Regional Office for Africa, Brazzaville, Republic of Congo; ^4^Public Health Consultant, Nairobi, Kenya; ^5^Assistant Regional Director's Office, WHO Regional Office for Africa, Geneva, Switzerland; ^6^Centre for Health Professions Education, Faculty of Health Sciences, North-West University, Potchefstroom, South Africa

**Keywords:** Sub-Saharan Africa, digital health, health systems strengthening, scoping review, digital health intervention

## Abstract

**Background:**

Digital Health Interventions (DHIs) refers to the utilization of digital and mobile technology to support the health system in service delivery. Over the recent years, advanced computing, genomics, and artificial intelligence are considered part of digital health. In the context of the World Health Organization (WHO) global strategy 2020–2025, digital health is defined as “the field of knowledge and practice associated with the development and use of digital technologies to improve health.” The scoping review protocol details the procedure for developing a comprehensive list of DHIs in Sub-Saharan Africa and documenting their roles in strengthening health systems.

**Method and Analysis:**

A scoping review will be done according to the Joanne Briggs institute reviewers manual and following the Preferred Reporting Items for Systematic Reviews and Meta-Analyses Extension for Scoping Reviews (PRISMA-ScR) checklist and explanation. The protocol has been registered at the Open Science Framework (OSF) database at https://osf.io/5kzq7. The review will include DHIs conceptualized/developed/designed, adapted, piloted, deployed, scaled up, and addressing health challenges in Sub-Saharan Africa. We will retrieve data from the global DHI repository-the WHO Digital Health Atlas (DHA)- and supplement it with information from the WHO eHealth Observatory, eHealth Survey (2015), and eHealth country profiles report. Additional searches will be conducted in four (4) electronic databases: PubMed, HINARI-Reasearch4Life, Cochrane Library, and Google Scholar. The review will also include gray literature and reference lists of selected studies. Data will be organized in conceptual categories looking at digital health interventions' distinct function toward achieving health sector objectives.

**Discussion:**

Sub-Saharan Africa is an emerging powerhouse in DHI innovations with rapid expansion and evolvement. The enthusiasm for digital health has experienced challenges including an escalation of short-lived digital health interventions, duplication, and minimal documentation of evidence on their impact on the health system. Efficient use of resources is important when striving toward the use digital health interventions in health systems strengthening. This can be achieved through documenting successes and lessons learnt over time.

**Conclusion:**

The review will provide the evidence to guide further investments in DHIs, avoid duplication, circumvent barriers, focus on gaps, and scale-up successful interventions.

## Introduction

The United Nations (UN) Member States agreed on seventeen (17) goals known as the Sustainable Development Goals (SDGs) to be achieved by the year 2030. The health sector goals are encompassed in the third SDG—“Ensure healthy lives and promote wellbeing for all at all ages” (1). The World Health Organisation (WHO) Global Action Plan for Healthy Lives and Wellbeing for all has identified seven accelerator themes to be implemented at country and regional levels. One of the accelerator themes is data and digital health recognized as key elements that will drive the achievement of the SDG 3 target. Digital health interventions offer an opportunity to address health system challenges, enhance coverage while maintaining the quality of service. Countries have a chance to meet the Sustainable Development Goals (SDGs) and Universal Health Coverage (UHC) targets by leap-frogging gaps in infrastructure. The interventions contribute to the goal of increasing availability and access to quality health services without occasioning financial hardships to the clients (2).

Successful digital health initiatives require a strategic delivery framework to coordinate implementation and monitor progress. The WHO vision of improving health for everyone through digital health solutions is outlined in the Global strategy on digital health 2020–2025 (3). The strategy expanded the concept of eHealth to include smart and connected devices, the Internet of Things, advanced computing, big data analytics, artificial intelligence including machine learning, and robotics. It was a culmination of a series of resolutions adopted by the World Health Assembly (WHA) starting in 2005. In May 2018, at the 71st WHA, WHO member states unanimously endorsed a resolution on digital health (4). In 2019, the WHO published a guideline on recommendations of digital interventions for health system strengthening in response to the Seventy-First World Health Assembly, member States resolution (5).

The field of digital health is dynamic with vast opportunities to support the needs of a health system. This has necessitated the development of a standardized language and classification to promote universal access to these interventions. The WHO has developed a classification framework based on the interventions' distinct function toward achieving health sector objectives. The guideline groups the interventions into four categories targeting: (1) Clients, (2) Health Care Providers, (3) Health System Managers, and (4) Data services (6).

Sub-Saharan Africa is a major contributor to the expanding and evolving field of digital health. The interventions in the region include mobile technologies, telemedicine collaborations, wearables and sensors, big data, and artificial intelligence amongst other interventions (7).

Digital health provides an opportunity to address health system challenges in the region if designed with consideration of the end-user, proper evidence, and a goal for large-scale roll-out. It is however important to remember that digital Health Interventions can enhance the functions of the health systems but cannot replace the fundamental health systems building blocks of (i) service delivery, (ii) health workforce, (iii) health information systems, (iv) access to essential medicines, vaccines, and technology (v) financing and, (vi) leadership/governance. This is due to limited access, acceptability and affordability of digital technology in populations (5).

The global eager to explore the full potential of digital health has experienced challenges. Many digital health interventions do not progress beyond the pilot stage. The duplication of efforts which result in resource wastage is a major challenge. Minimal documentation of evidence on the impact of these interventions on the health systems is a recurrent issue (3). To avoid further duplication and promote efficient use of limited resources, there is a need to explore, map, and summarize available interventions to highlight their potential, limitations and gaps as we aspire to strengthen health systems through digital health. Specifically, the objectives of the protocol are to:

1. List and classify digital health Interventions implemented in Sub-Saharan Africa over the last 10 years.
2. Categorize the digital health interventions to the appropriate development stage.
3. Outline the addressed health system challenges and align to the respective system categories.
4. Map the Digital health interventions to the target Health System Building block.
5. Highlight the potential, list the barriers, and identify gaps on utilization of DHIs in Health Systems Strengthening.

The review outcome will provide information to innovators, health system managers, and policy makers on where to allocate further investments in the use of DHI to strengthen health systems.

## Methods and Analysis

We will comprehensively search through DHI repositories, electronic databases, and gray literature to retrieve information on digital health interventions used in Sub-Saharan Africa. The review will be done according to the Joanne Briggs institute reviewers manual (8) and following Preferred Reporting Items for Systematic Reviews and Meta-Analyses Extension for Scoping Reviews (PRISMA-ScR) checklist and explanation (9). The protocol has been availed in an open-source platform–the Open Science Framework- at https://osf.io/5kzq7 (10). The study will use the Population, Concept and Context (PCC) format (Table 1) to align the methods and analysis with the research objectives.

**Table 1:** PCC study for eligibility of scoping review studies.

**Criteria**	**Determinants**
Population	Digital Health Interventions in Sub-Saharan Africa
Concept	The concept of interest in this review is use of Digital Health Interventions (DHIs) to strengthen the WHO Health System (HSS) building blocks. As such, the reviewed documentation must be aligned to strengthening one or more of the HSS-building blocks namely (i) service delivery, (ii) health workforce, (iii) health information systems, (iv) access to essential medicines, vaccines and Technology (v) financing, and (vi) leadership/governance
Context	The scoping review will include Digital Health Interventions conceptualized/developed/designed, adapted, piloted, deployed, and scaled up in the Sub-Saharan Africa over the last 10 years (2011–2021)

### Information Sources

The review will use peer-reviewed studies, policy documents, guidelines, government reports, and information from WHO-Digital Health Atlas, WHO eHealth Observatory, WHO eHealth Survey (2015), and WHO eHealth country profiles. The WHO Digital Health Atlas (DHA) will provide the initial list of the digital health interventions. The WHO eHealth Observatory, WHO eHealth Survey (2015) and WHO eHealth country profiles will provide in Supplementary Material. Additional searches will be conducted in four (4) databases: PubMed, HINARI-Reasearch4Life, Cochrane Library, and Google Scholar. We will use the keywords “Digital Health in Africa,” “Digital Health Interventions in Sub-Saharan Africa,” “Digital Health Tools in Sub-Saharan Africa,” and “Digital Health Technology in Sub-Saharan Africa.” A second search will include an analysis of the words contained in the title and abstract of retrieved papers, and of the index terms used to describe the articles. The review will also screen the reference lists for relevant studies. We will contact the DHI developers and/or authors where further information is needed.

Two independent reviewers will identify relevant documentation by screening the titles and abstracts against the proposed eligibility criteria (Table 2). The full-text documents will then be screened against the same eligibility criteria. Any disagreements on inclusion by the two independent reviewers will be resolved by a third reviewer in discussion with the first two reviewers. Pilot testing of the study selection process will take place and ensure 75% or greater agreement is achieved before undertaking formal screening and selection (11).

**Table 2:** Scoping review eligibility criteria.

**Question**	**Guiding response**
Does the documentation focus on a DHI?	Yes: Include No: Exclude documentations that do not report on implementation of a digital health Intervention.
Does the document report on implementation in Sub-Saharan Africa	Yes: Include No: Exclude all DHIs that are not conceptualized/developed/designed, adapted, piloted, deployed and scaled up in Sub-Saharan Africa
Was the intervention developed between January 2011- December 2021	Yes: Include No: Exclude all DHIs developed before 2011 or after 2021
Does the DHI address one or more health systems challenges	Yes: Include No: Exclude all DHIs that do not target any health system challenge as defined by WHO classification of Digital Health Interventions (6)

### Data Extraction

Data from the repository, the electronic databases and gray literature that fulfill eligibility criteria will be extracted into Excel. The data extraction tool will be adopted from the Joan Briggs institute Methodology guidance for scoping reviews (8) (Table 3). The tool will capture the key information from each document: First author/developer, year of publication/start of project country, purpose/aim/objective of the DHI, target end user, health system challenge addressed, system category, HSS building block, DHI development stage, implementation outcomes, barriers, opportunities, gaps, and any other key findings related to review question.

**Table 3:** Evidence source documentation and extracted information relevant to the scoping review.

**Data extraction tool**	
First author/developer	
Year of publication/start of implementation	
Country	
Purpose/Objective of DHI	
Stage of development	
Target end-user	
Health system challenge addressed	
System category	
HSS-Building block	
Barriers/Challenges	
Gaps	

### Data Analysis

The scoping review will utilize descriptive analysis. Data will be organized in conceptual categories looking at digital health interventions distinct function toward achieving health sector objectives, the target health system challenge and system category as per the WHO classification of Digital Health Interventions (6). The developmental stage of the technology will be classified as the WHO eHealth Survey 2015 (12). The interventions will be mapped to the appropriate Health System Strengthening (HSS) building blocks as per the WHO health systems Building Blocks Framework (13). The data will be tabulated as shown in Table 4 in Supplementary Material. The analysis will further document strengths and weaknesses, gaps, opportunities for building synergy, and current evidence for consideration in implementing digital health interventions in large scale. The results from the analysis will be described in relation to the research objectives and in the context of determining the extent of utilization of digital health interventions in health systems strengthening.

## Results

We will present a spatial and tabular distribution of identified Digital Health Interventions (DHIs) and the targeted Health System Strengthening (HSS) building blocks in Sub-Saharan Africa as per Supplementary Table 1. These will provide an overview of the concentration of DHIs in different countries and on specific HSS building blocks. Additionally, we will tabulate DHIs grouped by the HSS building blocks and include the analyzed parameters as shown in Supplementary Tables 2–7.

## Discussion

Scoping reviews form a basis of mapping concepts underpinning an area of focus through a guided search for evidence (14). Scoping review protocols contribute to an increasing need to synthesize and summarize research following a replicable design, implementation and reporting method (15). Growing evidence supports the contribution of digital health to efforts addressing health systems challenges. This is more so in Sub-Saharan Africa plagued by a strained health system attributed to inadequate human resources, inadequate budgetary allocations, and poor leadership and management in the health sector (16). The large-scale rollout of context-tailored digital interventions might improve health outcomes in the region due to the large tech-receptive young population (7).

The World Health Organization (WHO) has identified a list of priority DHIs that can be implemented in resource-limited settings to strengthen health systems. The list spans across technologies to improve vital statistic registration, interventions to facilitated supply chain management, platforms for telemedicine implementation, channels to communicate with clients, opportunities to improve access to medical records, knowledge repositories to support clinical decisions, and digital learning and training interventions for the health workforce (5). Furthermore, WHO has developed the digital implementation investment guide (DIIG) that provides a road map to facilitate the integration of DHIs into health programs. The guide elaborates the process of identifying ideal DHIs for specific contexts based on previous experiences and offers a systematic approach to the project cycle to increase the chances of successful integration of the DHIs (17).

Researchers have demonstrated the potential of DHIs to support health systems globally; the evidence generated is mostly on small scale and targets specific health focus areas. These include but are not limited to: the use of digital technology to support service delivery during disease outbreaks as witnessed during the Covid-19 pandemic (18–21), to offer sexual and reproductive health services (19, 22–25), in support of mental health interventions (19), to prevent and manage non-communicable diseases (26, 27) and to improve primary health care services (28). The health workforce has benefited from the enthusiasm for digital technology to enhance performance with positive outcomes (29–31). A health information system is a backbone for an evidence-based decision-making process at all levels of the health system. Digital technologies offer an opportunity to improve efficiency in the use of data to support health systems (32, 33).

The scoping review will identify and summarize digital health efforts aimed at alleviating health systems challenges in Sub-Saharan Africa. It will generate current evidence to guide future investments and highlight areas with the potential to address persistent challenges in the health systems.

### Strengths and Limitations

This protocol was developed following established methods of conducting scoping reviews. A review of existing policy and guideline documents was done to develop a standardized mapping strategy that can be replicated in other health focus areas. We however anticipate the following limitations:

First, the definition of digital health interventions is continuously evolving. We adopted WHO 2020–2025 definition in the study. Our inclusion and search strategy reflects this definition and might need an update in the future if the definition is significantly changed. Secondly, we did not have a framework to test the completeness of the WHO Digital Health Atlas repository and its complementary databases. Registering DHIs in the database is on voluntary basis. This creates a possibility of missing unregistered DHIs in Sub-Saharan Africa. Thirdly, few DHIs progress beyond the pilot stage. This results in minimal or no documentation of these digital health interventions in peer reviewed journals, repositories, and gray literature. Such DHIs will definitely be missed by the scoping review. To minimize the effect of these limitations, we constructed the search in a manner that increased comprehensiveness and inclusiveness while minimizing specificity.

## Conclusion

The scoping review will establish the current state of digital health in Sub-Saharan Africa and generate conclusions that will increase the efficiency of implementing DHIs with greater success. The results will identify future research areas to either generate robust evidence in support of scale-up or to adapt to context digital interventions with the greatest potential.

## Author Contributions

HK, DM, and VJ contributed to conception and design of the study. HK, DM, BD, VJ, CO, JN-O, and HCK wrote sections of the manuscript. HK consolidated the first draft of the manuscript. All authors contributed to the manuscript revision, read, and approved the submitted version.

## Conflict of Interest

The authors declare that the research was conducted in the absence of any commercial or financial relationships that could be construed as a potential conflict of interest.

## Publisher's Note

All claims expressed in this article are solely those of the authors and do not necessarily represent those of their affiliated organizations, or those of the publisher, the editors and the reviewers. Any product that may be evaluated in this article, or claim that may be made by its manufacturer, is not guaranteed or endorsed by the publisher.
