# Supplementary material for: Use of Digital Health Interventions in Sub-Saharan Africa for Health Systems Strengthening Over the Last 10 Years: A Scoping Review Protocol
Source: Front Digit Health. 2022 May 6;4:874251. doi: 10.3389/fdgth.2022.874251 (PMC9120370; doi:10.3389/fdgth.2022.874251)
Supplement: Supplementary file 1 [file Table_2.DOCX]

**Table 1**: Supplementary Material - Tabular distribution of Digital Health Interventions in sub-Saharan Africa and the targeted HSS building block

| Digital Health Intervention  (DHI) | Country of implementation | Start date | Overview of DHI | Targeted HSS building block |
| --- | --- | --- | --- | --- |
|  |  |  |  |  |
|  |  |  |  |  |
|  |  |  |  |  |

**Table 2:** Supplementary Material- Digital health interventions designed to strengthen service delivery in sub-Saharan Africa

| Targeted HSS- Building Block | Reference | Target User | | | | Stage of development | | | Addressed Health System Challenge | Aligned Systems Category |
| --- | --- | --- | --- | --- | --- | --- | --- | --- | --- | --- |
|  |  | Clients | Providers | Managers | Data services | Informal | Pilot | Established |  |  |
| Service Delivery | ---2011 |  |  |  |  |  |  |  |  |  |
|  | ---2015 |  |  |  |  |  |  |  |  |  |
|  | ---2021 |  |  |  |  |  |  |  |  |  |

**Table 3:** Supplementary Material- Digital health interventions designed to strengthen health workforce in sub-Saharan Africa

| Targeted HSS- Building Block | Reference | Target User | | | | Stage of development | | | Addressed Health System Challenge | Aligned Systems Category |
| --- | --- | --- | --- | --- | --- | --- | --- | --- | --- | --- |
|  |  | Clients | Providers | Managers | Data services | Informal | Pilot | Established |  |  |
| Health Workforce | ---2011 |  |  |  |  |  |  |  |  |  |
|  | ---2015 |  |  |  |  |  |  |  |  |  |
|  | ---2021 |  |  |  |  |  |  |  |  |  |

**Table 4:** Supplementary Material- Digital health interventions designed to strengthen health information system in sub-Saharan Africa

| Targeted HSS- Building Block | Reference | Target User | | | | Stage of development | | | Addressed Health System Challenge | Aligned Systems Category |
| --- | --- | --- | --- | --- | --- | --- | --- | --- | --- | --- |
|  |  | Clients | Providers | Managers | Data services | Informal | Pilot | Established |  |  |
| Health Information System | ---2011 |  |  |  |  |  |  |  |  |  |
|  | ---2015 |  |  |  |  |  |  |  |  |  |
|  | ---2021 |  |  |  |  |  |  |  |  |  |

**Table 5:** Supplementary Material- Digital health interventions designed to strengthen access to essential medicines, vaccines, and technology in sub-Saharan Africa

| Targeted HSS- Building Block | Reference | Target User | | | | Stage of development | | | Addressed Health System Challenge | Aligned Systems Category |
| --- | --- | --- | --- | --- | --- | --- | --- | --- | --- | --- |
|  |  | Clients | Providers | Managers | Data services | Informal | Pilot | Established |  |  |
| Access to Medicines, Vaccines, and Technology | ---2011 |  |  |  |  |  |  |  |  |  |
|  | ---2015 |  |  |  |  |  |  |  |  |  |
|  | ---2021 |  |  |  |  |  |  |  |  |  |

**Table 6:** Supplementary Material - Digital health interventions designed to strengthen health financing in sub-Saharan Africa

| Targeted HSS- Building Block | Reference | Target User | | | | Stage of development | | | Addressed Health System Challenge | Aligned Systems Category |
| --- | --- | --- | --- | --- | --- | --- | --- | --- | --- | --- |
|  |  | Clients | Providers | Managers | Data services | Informal | Pilot | Established |  |  |
| Health Financing | ---2011 |  |  |  |  |  |  |  |  |  |
|  | ---2015 |  |  |  |  |  |  |  |  |  |
|  | ---2021 |  |  |  |  |  |  |  |  |  |

**Table 7**: Supplementary Material- Digital health interventions designed to strengthen leadership/governance in sub-Saharan Africa

| Targeted HSS- Building Block | Reference | Target User | | | | Stage of development | | | Addressed Health System Challenge | Aligned Systems Category |
| --- | --- | --- | --- | --- | --- | --- | --- | --- | --- | --- |
|  |  | Clients | Providers | Managers | Data services | Informal | Pilot | Established |  |  |
| Leadership and Governance | ---2011 |  |  |  |  |  |  |  |  |  |
|  | ---2015 |  |  |  |  |  |  |  |  |  |
|  | ---2021 |  |  |  |  |  |  |  |  |  |
