# Supplementary material for: Use of Digital Health Interventions in Sub-Saharan Africa for Health Systems Strengthening Over the Last 10 Years: A Scoping Review Protocol
Source: Front Digit Health. 2022 May 6;4:874251. doi: 10.3389/fdgth.2022.874251 (PMC9120370; doi:10.3389/fdgth.2022.874251)
Supplement: Supplementary file 2 [file Table_1.DOCX]

**Table 4:** Supplementary Material- Data tabulation

| HSS- Building Block | Reference | Target User | | | | Stage of development | | | Health System Challenge | Systems Category |
| --- | --- | --- | --- | --- | --- | --- | --- | --- | --- | --- |
|  |  | Clients | Providers | Managers | Data services | Informal | Pilot | Established |  |  |
| Service Delivery | ---2011 |  |  |  |  |  |  |  |  |  |
|  | ---2015 |  |  |  |  |  |  |  |  |  |
|  | ---2021 |  |  |  |  |  |  |  |  |  |
| Health Workforce | ---2011 |  |  |  |  |  |  |  |  |  |
|  | ---2015 |  |  |  |  |  |  |  |  |  |
|  | ---2021 |  |  |  |  |  |  |  |  |  |
